# Prospects of Microfluidic Technology in Nucleic Acid Detection Approaches

**DOI:** 10.3390/bios13060584

**Published:** 2023-05-27

**Authors:** Zilwa Mumtaz, Zubia Rashid, Ashaq Ali, Afsheen Arif, Fuad Ameen, Mona S. AlTami, Muhammad Zubair Yousaf

**Affiliations:** 1 KAM School of Life Sciences, Forman Christian College University, Ferozpur Road, Lahore 54600, Pakistan; 2 Pure Health Laboratory, Mafraq Hospital, Abu Dhabi 1227788, United Arab Emirates; 3 State Key Laboratory of Virology, Center for Biosafety MegaScience, Wuhan Institute of Virology, Chinese Academy of Sciences, Wuhan 430071, China; 4 Karachi Institute of Biotechnology and Genetic Engineering (KIBGE), University of Karachi, Karachi 75270, Pakistan; 5 Department of Botany and Microbiology, College of Science, King Suad University, Riyadh 11451, Saudi Arabia; 6 Biology Department, College of Science, Qassim University, Burydah 52571, Saudi Arabia

**Keywords:** nucleic acid detection, microfluidics, portable devices, molecular diagnostics, isothermal amplification, lab on chip, nucleic acid testing

## Abstract

Conventional diagnostic techniques are based on the utilization of analyte sampling, sensing and signaling on separate platforms for detection purposes, which must be integrated to a single step procedure in point of care (POC) testing devices. Due to the expeditious nature of microfluidic platforms, the trend has been shifted toward the implementation of these systems for the detection of analytes in biochemical, clinical and food technology. Microfluidic systems molded with substances such as polymers or glass offer the specific and sensitive detection of infectious and noninfectious diseases by providing innumerable benefits, including less cost, good biological affinity, strong capillary action and simple process of fabrication. In the case of nanosensors for nucleic acid detection, some challenges need to be addressed, such as cellular lysis, isolation and amplification of nucleic acid before its detection. To avoid the utilization of laborious steps for executing these processes, advances have been deployed in this perspective for on-chip sample preparation, amplification and detection by the introduction of an emerging field of modular microfluidics that has multiple advantages over integrated microfluidics. This review emphasizes the significance of microfluidic technology for the nucleic acid detection of infectious and non-infectious diseases. The implementation of isothermal amplification in conjunction with the lateral flow assay greatly increases the binding efficiency of nanoparticles and biomolecules and improves the limit of detection and sensitivity. Most importantly, the deployment of paper-based material made of cellulose reduces the overall cost. Microfluidic technology in nucleic acid testing has been discussed by explicating its applications in different fields. Next-generation diagnostic methods can be improved by using CRISPR/Cas technology in microfluidic systems. This review concludes with the comparison and future prospects of various microfluidic systems, detection methods and plasma separation techniques used in microfluidic devices.

## 1. Introduction

It is important to diagnose infections that are caused by pathogens at their earliest stage, as they are transmitted between animals and humans by inoculation from different media, such as water and air [1,2,3]. On the other hand, pathogen detection is not only important in the health care sector, but it is also important regarding safety issues in areas such as food, water or production facilities [4].

Therefore, it is important to detect them so that treatments can be initiated properly. Along with treatment, its prevention is also necessary, which calls for a low-cost, rapid, on-site and accurate diagnosis, especially in areas that are devoid of resources and have severe and prevalent infections [5]. In this perspective, microfluidic technology that combines material science, nanotechnology and micro electro mechanical systems for specified fluidic manipulations provide opportunities for point of care testing (POCT) devices for the detection of pathogens and diseases. Amplification and sequencing strategies such as PCR and NGS are molecular diagnostic techniques that have demonstrated outstanding sensitivity and specificity. In comparison to these conventional diagnostic methods, portable microfluidic devices employ miniaturized devices that range from large laboratory analyzers to disease specific screening platforms by which on-site testing can be performed. The reason behind the fast expansion of microfluidics is the development of small-scale components that allow the handling of materials on a microscale level [6,7]. Molecular diagnosis is the most dependable, accurate and sensitive way for a disease diagnosis among the various available techniques [8].

Although endogenous RNA can be detected using microfluidic devices, their use in isolating purified viral RNA from blood samples has received less attention. This is probably because extra cellular vesicles would have to be isolated to identify these biomolecules present there. Extracellular RNA is structurally more unstable than DNA, making its detection more difficult, especially in integrated miniaturized devices. Since plasma contains both cell free double stranded DNA (dsDNA) and RNA, sample preparation methods before nucleic acid detection call for cellular lysis, filtration and centrifugation, whereas the extraction and purification of nucleic acid can be performed by various methods such as dielectrophoresis, membrane filtration, microbeads and microfluidic channels. The first and most significant benefit of microfluidic technology is its low energy consumption, high-throughput applications, spatial temporal precision, portable systems and quick prototyping [9].Traditional techniques for directly detecting nucleic acid include polymerase chain reaction (PCR), the enzyme linked immunosorbent assay (ELISA), isothermal amplification, clustered regulatory interspaced short palindromic repeats (CRISPR) and aptamer- based detection, whereas in direct methods of nucleic acid detection involve the detection of antibodies generated by patients in response to infection. Although the indirect method has many applications, it has some drawbacks, such as low specificity and accuracy [10,11]. In spite of the fact that the detection of nucleic acid is required in most of the fields, healthcare, biodefense, biomonitoring and environmental monitoring are the four major sectors that require more precise nucleic acid detection [12].

Various biosensors have been developed for determination of the quantity and type of a biomolecule by modifying the mechanical, chemical or electrical properties of the sensing element to produce a quantifiable signal. Most of the nucleic acid testing methods rely on DNA probes, which are used to hybridize with target nucleic acids. The tagged DNA is joined to the detector molecule and has a length of 30–40 nucleotide bp. Evaluation of the nucleic acids is important, but this technology has certain drawbacks. Particularly, only a few genes can be probed in this process. The genes can be detected on biochips and biosensors which ensures the presence of biomolecules such as enzymes, proteins and nucleic acids. However, biochips can be divided into three categories: lab-on-a-chip, biochips for analyzing genes and biochips for the protein analysis. In this review, our focus will be on lab-on-a-chip technology, which uses miniature devices to perform laboratory operations on a small scale. The perks of this method include a shorter analysis time, less chemical waste generation, lower costs and compact instrumentation with the ability to analyze samples immediately without the need for a laboratory [13].

Here, we have discussed the role of microfluidic technology in the detection of nucleic acid regarding health and safety. Next, we cover the materials for making microfluidic devices and a comparison of plasma separation methods in microfluidic systems. Eventually, several detection strategies are compared, followed by non-isothermal and isothermal means of amplification. Furthermore, the prospects and challenges faced by microfluidic technology in diagnostics are debated at the end.

## 2. Microfluidic Technology and POCTs

### 2.1. Significance of POCT in Diagnostics

The POCT platforms controlled by microfluidics employ portable and small devices for conducting on-site sampling in contrast to conventional diagnostics.

**Figure 1:**
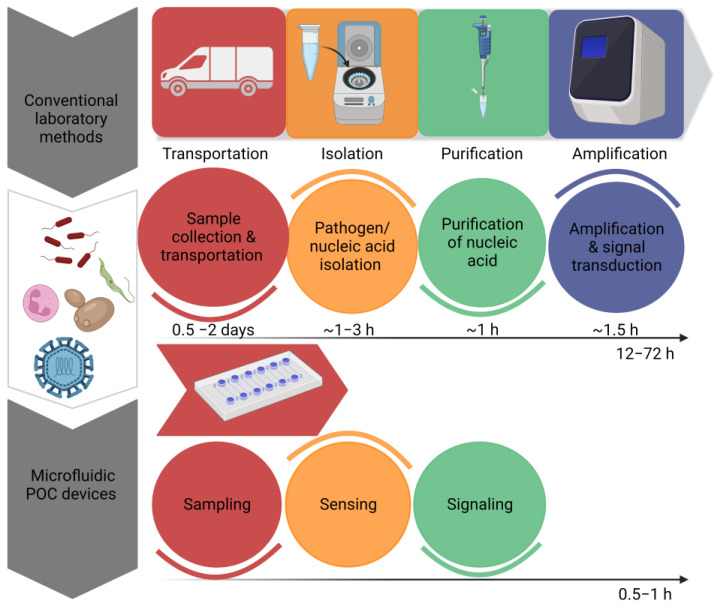
Comparison of the estimated time required to perform a diagnostic test by traditional methods and microfluidic POCT devices.

By omitting time-consuming sample preparation stages, the innovations have enabled the development of quick signal amplification with increased sensitivity. Recent developments in molecular diagnostics have resulted in the development of a disease diagnosis based on nucleic acids [14]. However, sample preprocessing, the amplification of nucleic acids and signal readouts are the three main steps in nucleic acid testing. These devices offer a preliminary screening of diseases in a non-laboratory setting; however, they cannot replace sensitive laboratory tests. By recognizing the significance of POCT devices, the World Health Organization has set a criterion for the evaluation of clinical diagnoses. According to the WHO, POCT devices have been given an acronym of ASSURED, which means that the test must be ‘Affordable, Sensitive, Specific, User- friendly, Rapid and robust, Equipment free & Deliverable’ [15]. Figure 1 Shows the efficiency of microfluidic systems by estimating time required to perform a diagnostic test by traditional methods and microfluidic POCT devices. “Created with BioRender.com”.

### 2.2. Microfluidic POCT Devices

Microfluidics is a suitable technique for designing complex bioaffinity sensors due to its ease of manufacture, small sample and reagent amounts, short reaction time and suitability for result observations. The primary purpose of microfluidics is to accelerate chemical reactions in tiny volumes, where there is more atomic exposure during the reaction in micrometer scales of microfluidic channels where the surface to volume ratio is several orders greater than in bulk systems. When the length scale is shortened, the diffusion process proceeds with a time scale of d ~/2τlD, where D is the sample’s diffusion coefficient. As a result, quicker chemical and biological reaction rates are attained. In addition, droplet microfluidics divides the continuous flow of bulk solutions into discrete quantities in the form of droplets. A frequency of several kilo hertz can be used to sort droplets, enabling high-throughput processes and preventing cross-contamination [16].

In POCT devices, the microfluidic platforms require a system for delivering samples and reagents, a mechanism for mixing and moving fluids and detectors for assessing microanalytical operations [15]. Cell lysis is the first and most crucial step in sample preparation for nucleic acid testing. However, chemical and mechanical cell lysis are the most frequently used methods to accomplish this step [17]. For the on-chip testing of nucleic acid, chips can be divided into three parts for sampling, sensing and signaling. The basic modules of microfluidic devices include micropumps, microvalves, micromixers, droplet generators, separators, traps, cell cultures and gradient generators, whereas six commonly used connection methods include tubing connection, O-ring/gasket connection, LEGO connection, Luer connection and plasma and adhesive connections. Comparatively, modular microfluidics has advantages over integrated microfluidics in terms of free selection of the arrangement and number scheme of the modules, optimization of individual steps for analysis and the adjustment of local region conditions of the system and for various types of experimental objects [18].

In addition to its stability, biocompatibility and adaptability, silicon is a suitable material for the isolation of nucleic acid [19]. In POCT devices, porous materials have been utilized to extract nucleic acid for diagnostic purposes. A commercially accessible solid phase nucleic acid extraction technique called Finders Technology Associates (FTA) cards are used to manufacture POCT devices that also amplify nucleic acid. FTA cards can be coupled with a Loop mediated isothermal Amplification (LAMP) cassette for detection of the Human Immunodeficiency Virus (HIV) [16,17]. Microfluidic devices require sample volumes less than 100µL for downstream detection purposes [20]. Three types of reactors have been used for microfluidics PCR. For a microfluidic PCR, the thermal cycling time is calculated in minutes, and the size of the reactor is required in μL, whereas a microfluidic PCR is defined as γ [21]. The first type of reactor is termed as a stationary reactor, forced continuous flow PCR is the second type of reactor and the third type of reactor is composed of a free heat deportation system [21,22,23,24].

### 2.3. Materials for Microfluidic Devices

Paper, polymer and inorganic materials are the main three types of materials used to interface microfluidic devices. Initially, glass and silicon materials were used. Thermoplastics and elastomers are two subcategories of polymeric substances that were introduced later. Inorganic materials include cofired low-temperature ceramics and Vitro ceramics. Paper microfluidics has been considered as a slightly distinct technology from polymeric and inorganic microfluidics. Before choosing the material type for a microfluidic system, it is important to consider three factors: application, degree of integration and function. Some other physical properties that must be considered before choosing a material include air permeability, flexibility, nonspecific adsorption, electrical conductivity, cellular compatibility and optical transparency [25].

Glass, polymers and silicon are frequently used components for designing a microfluidic device. Silicon has been widely used in the semiconductor industry due to its well studied properties and developed fabrication methods. Most of the silicon-based devices have a layer of SiO_2_ thermally grown on their surface, especially the sensors that rely on electrically based detection methods. Glass is an ideal material for biosensors that use optical detection techniques such as fluorescence or surface plasmon resonance (SPR) because of its qualities, such as chemical stability and transparency. One of the most popular types of glass is soda lime; however, it has a lot of contaminants, such as aluminum. Microfluidic devices typically employ Pyrex glass or borosilicate, which are more expensive than other forms of glass. Quartz or fused silica are also used due to their best optical properties. However, the cost of these materials is considerably higher, but the use of glass in microfluidic devices faces the challenge of channel sealing due to the difficulty in bonding device layers. Mostly, high temperatures or large electric fields are needed to meet device bonding requirements.

#### Paper-Based Multiplexed Detection

Primarily, three methods are used to implement paper- based multiplex detection: spatial separation of detection sites; regionally employing distinct channel components or using various labels such as enzymes, redox compounds and beads or colors. Regional separation is a key feature of paper–based microfluidic chips, providing a high degree of multiplexing, as well as better testing efficiency and versatility. An innovative concept for multiplex paper-based detection is target responsive DNA hydrogel. Similarly, a paper-based chip was designed to detect numerous targets simultaneously by using a target-responsive flow controller made of an aptamer cross linked hydrogel, a target responsive hydrogel having a cross linked aptamer on paper to control the fluid flow and signal readout [26,27].

### 2.4. Microfluidic Approaches for Plasma Separation in POCT Devices

Active and passive techniques are used for the separation of serum/plasma from blood in a microfluidic system. In a passive approach, blood cells are moved by passive means as they pass through plain or bead filled tubes. These techniques include hydrodynamic focusing, deterministic lateral displacement, sedimentation and filtration. Active techniques such as acoustophoresis or dielectrophoresis, on the other hand, exert forces on cells to move as they pass through channels of the microfluidic system.

#### Limitations of Active and Passive Methods

Hemolysis has been demonstrated to interfere with several metabolites assays due to releasing hemoglobin into the blood. Every microfluidic separation technology has specific limitations, such as the lengthy working durations of devices based on the principle of sedimentation and the difficult manufacturing processes of devices that use external forces. With a few notable exceptions, most microfluidic devices only recover trace amounts of serum/plasma from diluted blood samples, which may not be sufficient for detection purposes. However, sometimes, these systems need some kind of extra hardware, such as a motor, syringe pump or other external hardware, to control the flow of a fluid, which makes them more complex. Some approaches of microfluidic systems for separation of serum/plasma from blood have been discussed in Table 1.

However, an easy-to-integrate extraction method has been developed known as Isotachophoresis (ITP), which is a nucleic acid extraction method based on the separation of analytes because of their mobility under the application of pressure in an electric field. This technique can be used with small volumes that can be further automated for purification and amplification steps. Additionally, ITP compatible with the CRISPR/Cas system integrated with a microfluidic device has been designed for the detection of viral RNA in 35 min [35].

### 2.5. Amplification Methods

#### 2.5.1. Non-Isothermal Amplification

A microfluidic platform has been developed that has a special capacity to amplify PCR templates in an affordable thermoplastic structure with a cycle time of 14 s and complete reactions in 8.5 min [36]. Similarly, for PCR amplification, a disc shaped microfluidic device with independent components of a heating system, a small aluminum plate and diaphragm valve has been designed. This device pumps liquids using a centrifugal force and demonstrates robust, leak-free, reversible and valves actuation at thermally stable conditions during PCR amplification in a fully automated way [37]. A microfluidic thermalization system was developed for ultrafast PCR with a total reaction time of <8 min. The process relies on the movement of a heated solution in a chip to thermally activate the PCR chamber through diffusion [38]. High speed thermalization of this system allowed it to conduct a sharp melting curve analysis in addition to running PCR reactions within a few minutes. Moreover, due to its low cost and high efficiency, researchers have suggested the integration of this microfluidic system in POCT devices [39,40].

#### 2.5.2. Isothermal Amplification

Although many PCR-based microfluidic devices have been developed, the accurate control of thermal cycling and integration of a heater are still major issues with POCT devices in some developing countries. Therefore, to address these issues, scientists have developed isothermal amplification processes that use an amplification technique without a thermocycler. The isothermal amplification processes used for diagnostic purposes are discussed below.

##### Loop-Mediated Isothermal AMPlification (LAMP)

In this approach, a DNA polymerase with strand displacement activity is used along with two sets of specially designed primers known as the forward inner primer (FIP) and backward inner primer (BIP). Four more primers are also used to increase the specificity of the amplification. Amplification is performed within an hour of target sequence recognition. In LAMP, 60 to 65 °C temperate is required to carry out amplification, and the product is easily detected by the naked eye due to the production of white precipitates that are pyrophosphate by products [41]. One such device has been developed for the amplification and detection of Hepatitis B Virus (HBV) [42,43]. However, this technique is not suitable for the amplification of short genes [44]. Advancements have been made to reduce the reaction volume in microfluidic–integrated LAMP devices or microLAMPs (μLAMP) [42].

##### Helicase Dependent Amplification (HDA)

After analyzing the properties of helicases, researchers have linked polymerase with helicases and a few additional proteins for nucleic acid amplification. HDA mimics the denaturation step used in traditional PCR by using DNA helicase activity to separate double stranded DNA (dsDNA). DNA polymerase initiates replication following this separation step, where chemical energy is used for the formation of a replication fork. Two target sequence-specific primers are used that anneal to the 3′ end of both strands of single stranded DNA (ssDNA) [45]. The microfluidic chip for HDA has been integrated with a small scale solid phase extraction column for the isolation of DNA [46]. By integrating HDA on the strip, a new technique was adopted to identify the source of ruminant fecal contamination [47].

##### Rolling Circle Amplification (RCA)

This method makes use of a special DNA polymerase with strand displacement activity to amplify circular DNA. Amplification is performed at a constant temperature to produce ssDNA with tandem repeats of the circular template. Both linear and exponential methods are used to perform RCA. The amplification efficiency of this method depends on the requirement of circular and small ssDNA as a template. However, most of the DNA templates required for diagnosis are single stranded DNA. To avoid this issue, a special type of probe has been designed called pad lock probes that circularize following hybridization with the target sequence [48]. The single molecular amplification of DNA by droplet microfluidic hyper branched RCA has been developed. Nowadays, droplet digital RCA systems have been designed for the sensitive and rapid detection of cancerous cell-derived extracellular vesicles [49]. An electrochemical aptasensor (ultrasensitive) was developed for multiple exosome biomarker detection in breast cancer, which was based on dual RCA [50].

##### Multiple Displacement Amplification (MDA)

In this amplification approach, amplification of the whole genome is carried out by generating many amplicons from a small number of molecules of DNA. No thermal cycling is required for the reaction, because the primers are randomly exonuclease resistant, and there is a strand displacement activity. The use of nanoliter size d microfluidic reactors and a cell sorting device for the isolation of specific individual cells can increase the specificity of MDA, whereas reducing the reaction volumes from µL to nl reduces the nonspecific amplification [51,52,53]. The technique has been expanding rapidly because of its ability to differentiate defective DNA. MDA has been used in the diagnosis of tuberculosis (TB) by performing the MDA of DNA in a two-step process [54].

##### Recombinase Polymerase Amplification (RPA)

A complex of DNA polymerase, DNA–binding proteins and a recombinase are used to amplify DNA at 37 °C. This complex is used for dsDNA scanning, primer binding and non-template strand displacement. Single stranded DNA–binding proteins (SSBP) stabilize the displaced strand, and recombinase makes the 3′ end accessible for DNA polymerase.

A simple and low-cost method was used for the development of a microfluidic chip for RPA by combining the dry film resist technique and direct wafer bonding [55]. As the RPA reaction is carried out at or near to the room temperature range, premixing of the sample with an initial reagent may proceed the reaction without compartmentalization. Despite its little applications in clinical sectors, authentications have been made regarding its use in the detection of viruses, bacteria, protozoa and human pathogens [56,57].

##### Nucleic Acid Sequence Based Amplification (NASBA)

NASBA is a technique for amplifying ssDNA or RNA sequences at 41 °C without any requirement of a denaturation step. Due to which, dsDNA templates cannot be amplified by this method. The preferable templates for carrying out NASBA are mRNA, genomic RNA and rRNA. Two RNA sequence specific primers and three enzymes are used in this technique. These enzymes are T7 DNA-dependent RNA polymerase (DdRp), avian myeloblastosis virus reverse transcriptase and RNase H. A microfluidic device has been developed for the immuno-NASBA detection of water borne pathogens. This lab on a chip device relies on the utilization of different antibodies for the recognition of various targets in a single step procedure [58]. Table 2 shows a comparison of various isothermal amplification methods based on template requirement, estimated time, pairs of primers and approximate melting temperature.

### 2.6. Strategies for Nucleic Acid Testing

Nucleic acid testing (NAT) offers many advantages over conventional immunoassays due to better sensitivity and specificity. The most used laboratory techniques for nucleic acid detection include PCR, real-time PCR and reverse transcription PCR [67]. A membrane embedded in a microfluidic chip binds DNA to isolated from the cell. The nucleic acid is further amplified at a constant temperature, and the amplified product containing a fluorophore reporter is detected by a detector (CCD detector or photodiode) [68]. To carry NAT on microfluidic POCT, the device must be fabricated when it is in its initial manufacturing phase. The continually used fabrication techniques for microfluidic systems are printing and cutting, photolithography and molding [69]. Bubble formation, cross contamination and reagent evaporation are some common issues with DNA amplification in microchannels, which can be reduced by using polydimethylsiloxane to seal the channels (PDMS) [70].

**Figure 2:**
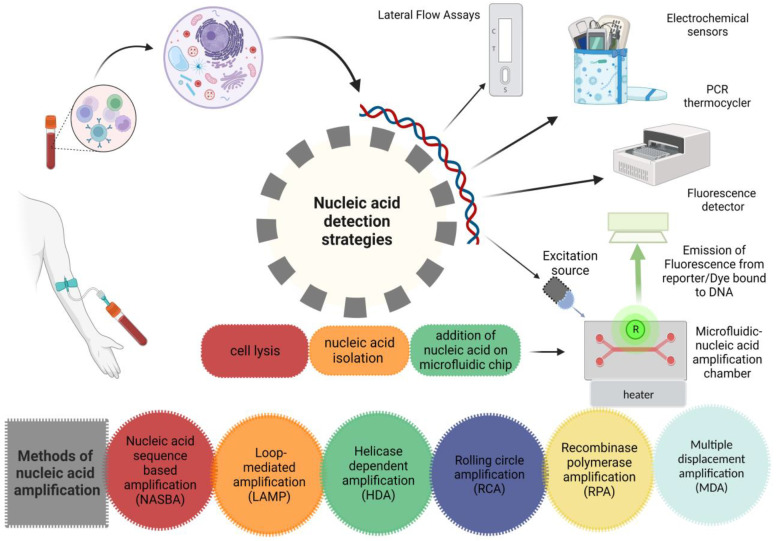
Nucleic acid-based detection strategies.

It is not surprising to say that more sensitive tests are usually less specific. The specificity of NAT is relatively high, i.e., more than 99.0%, as compared to other methods. Therefore, the utilization of NAT in a low prevalence population will result in false-positive results. In a test with 99.5% specificity, 0.5% of the positive results will be false positives. If there is 1.0% positivity of the results in a population, only 50% of the patients will be positive. Cross-checking the specimen with an alternative assay can increase the specificity of the test [71]. The development of novel diagnostic tests must always include studies on reproducibility within and between laboratories. High sensitivity and specificity cannot coexist with only modest test retest agreement. Possible causes of inconsistent findings for NAATs include false-positive hybridization during the detection phase for instance, nonspecific priming in the amplification phase, amplicon contamination and the presence of inhibitors [72]. Figure 2 Shows various strategies including microfluidic systems and other traditional methods for detection of nucleic acid. “Created with BioRender.com”.

#### 2.6.1. Paper-Based Microfluidics

Conventional lateral flow assays (LFAs), which are used for the risk assessment, diagnosis and treatment of both infectious and non-infectious diseases, may be considered the simplest and earliest type of microfluidic devices. Since the advancement of mobile phone’s optical imaging technology, the quantitative analysis of LFA at POCT is now performed more accurately. A more precise quantitative measurement of LFA is now possible because of the improvements in optical imaging technologies of mobile phones. Due to the efficiency of nanomaterials in signal transduction and better stability, AuNPs, carbon nanoparticles and quantum dots are now essential components of LFA. Microchannels provide depth to paper-based microfluidic (µPAD) platforms, but the linear fluidics used in LFA limits the functional parameters and analytical efficacy of PADs. Numerous methods have been used for patterning paper that include photolithography, printing, laser treatment and plasma treatment. A recent innovation in paper-based devices includes the origami analytical device (oPAD), also known as 3D µPAD, developed by a single patterning process and is simple to fold and align without the use of cellulose powder in between layers [73].

The nucleic acid lateral flow assay (NALFA) has drawn attention in POCT fields due to its ease of implementation, rapidity and less requirement of equipment, which makes them a well suited candidate in environmental monitoring, food authentication and diagnosis in resource limited areas. The integration of isothermal amplification on LFA increases the sensitivity of the assay. The two frequently used isothermal amplification strategies include RPA and LAMP [74]. A sensitive NALFA was developed, which, at lower concentrations of the target, gave little difference in capturing the efficiency between the capture and detector probe-conjugated AuNPs, and when the concentration was increased, the capturing efficiency of the capture and detector probe conjugated gold nanoparticles (AuNPs) lagged the AuNP detector probe conjugates [75]. Bio-barcode designing was another approach adopted for the development of NALFA [76]. One approach that can be adopted to prevent the spreading of reagents during the deposition of narrow microfluidic control and the test line is the utilization of inkjet printers. Filter paper based LFAs fabricated with inkjet printing technology offer a confined control and test line. Despite their challenges, inkjet-printed µPADs are still considered attractive. The formulation of ink is the main key to printing, which requires various compositions of inks [77].

#### 2.6.2. Polymer-Based Microfluidics

Recent advancements in polymer-based microfluidics have drawn considerable interest for the detection of infectious diseases in particular, an approach based on CRISPR/Cas13a that uses the amplification-free smart phone microscopy method on PDMS to identify the viral RNA of ~100 copies L1 in thirty minutes [78]. Due to its distinct benefits, such as higher compatibility with biomolecules, higher transparent observations, higher permeability of gas for diffusion and relatively simple manufacturing method of lithography, polydimethylsiloxane (PDMS) is particularly employed frequently. Similarly, polymethylmethacrylate (PMMA) has the benefits of being reasonably priced and having acceptable mechanical and optical characteristics [77,79].

#### 2.6.3. CRISPR-Based Microfluidic Systems

Next generation diagnostic methods can be improved with precise CRISPR/Cas technology by taking advantage of the benefits of microfluidics. This system has multiple benefits, which include less cost, less consumption of the reagents, quick turnaround time, high specificity and ability to incorporate a multiple detection approach. Microfluidic-based CRISR/Cas systems are mainly based on the utilization of Cas9, Cas13a and Cas12a proteins. Table 3 Shows comparison of microfluidic biosensors on the basis of their manufacturing materials, types, detecting pathogens, bioanalytes, amplification methods, detection methods, ability to perform multiplexing, sensitivity, limit of detection, limitations and prospects.

#### 2.6.4. Digital Microfluidics

In a magnetic barcode assay, the mycobacterial gene was amplified by PCR and caught on polymer beads modified with complementary DNA by a specific sequence, labeled by magnetic nanoparticles in a miniaturized nuclear magnetic resonance (NMR) chamber and then evaluated by micro-NMR. Miniature tools such as mini microscopes, a portable mass spectrometer and systems compatible with smart phones have additionally made it easier to identify and analyze POC testing and various encoded assays [80]. Digital microfluidics (DMF), with a continuous flow, has the benefit of less reagent consumption, quick responses and carrying out multiple processes simultaneously. The Wheeler group created the Measles Rubella Box (MRBox), a 4-kg instrument made up of a portable control system and inexpensive DMF cartridges, to extend the clinical use of DMF-based biosensors [81].

**Table 3:** Comparison of polymer-based, paper-based, quartz/glass and some other microfluidic biosensors.

Biosensors	Biosensor Type/ Pathogen	Bioanalyte/Amplification Method		Detection Method/ Multiplex (Y/N)	Sensitivity &Detection	Limitations/Future Prospects	Ref.
Polymer-based devices							
PDMS-based nanoarray	Cas 12a/HBV, HPV-16, HPV-18	Nucleic acid(NA)/Amplification freemethod		Surface-enhanced Raman scattering/ Y	Sensitivity: 1 aM Time: 20 min	High cost, only for DNA targets Optimization strategies should be taken in future to increase sensitivity of targets and strength of SERS signal. By reducing Limit of detection and assay time, overall performance of system can be improved shortening the assay time and reduction of the detection limit may also improve the system. Several biomarkers including proteins or RNA can be detected by utilization of Cas13a for RNA targets and aptamers for proteins	[81]
POCKET (POC kit for the full test)	Microfluidic PDMS-based/Mutational analysis of Southeast Asia thalassemia	DNA/RPA		Colorimetric/ Y	Sensitivity: <103 copies/mL Time: <2 h	N/A In future, the variety of sample types will be increased to include those samples that are challenging to prepare, such assputum and feces. An independent and power free method can be developed to control reagent loading procedure in a better way Prevention of cross contamination from positive samples	[8]
Paper-Based devices							
Paper-Based device		CRISPR/Ca/SARS-CoV-2	NA/RT-RPA	Colorimetric/ N	Sensitivity: LOD 100 copies Time: 1 h	N/A Issue of competitive hybridization can be avoided by designing two types of probes for individual identification of each target By optimizing nitrocellulose membrane’s pore size, flow rate of the strip or running buffer can be improved to ensure a better binding efficiency	[82]
SHERLOCKv2		Cas13, Cas12a and Csm/Dengue, Zika virus	NA/RPA	Fluorescence/Y	Sensitivity: aM Time: 1 h	N/A Solution and colorimetric-based readouts and multiplex lateral flow assays containing multiple test strips for different targets can be developed in future	[83]
CRISPR-based microfluidic LFA chip		Cas12a/ SARS-CoV-2	NA/RT-RPA	Colorimetric/ N	Sensitivity: 100 copies Time: <2 h	N/A Fully integrated molecular detection platform by adding nucleic acid extraction module to microfluidic chip Incorporation of phase changing material into heater case to control temperature	[84]
CASLFA (CRISPR/Cas9-mediated lateral flow assay)		Cas9/SARS-CoV-2, African swine fever virus (ASFV), Listeria monocytogenes, genetically modified organisms (GMOs)	NA/RPA	Colorimetric/ N	Sensitivity: 100 copies Time:1 h	A number of separation stages are still needed to complete the process Future research may combine microfluidic technology with the developed system to integrate extraction, amplification and detection on a single platform For multiplexing, primers with different labels and variety of test lines will enhance the feasibility of CASLFA to detect multiple targets simultaneously.	[85]
MiSHERLOCK (minimally instrumented SHERLOCK)		Cas-12a/SARS-CoV-2	RNA/RPA	Fluorescence/Y	Sensitivity: 1240 cp/mL Time: 60 min	Only a few COVID-19 patient samples were examined Owing to lack of resources, SARS-CoV-2 variants could not be tested Screening and diagnosis of disease variants	[86]
SHINE		Cas13a/SARS-CoV-2	NA/RPA	Fluorescence/ N	Concentration: 10 copies L1 Time: 50 min	Inconsistent measurements with RT-qPCR Lyophilization of reagents would facilitate assay preparation and dissemination while enabling shelf stable testing	[87]
HUDSON(heating unextracted diagnostic samples to obliterate nucleases)		Cas 13-based/Dengue virus, Zika virus, resistance genes, bacteria	NA/RPA	Fluorescence and colorimetric/N	Sensitivity: aM, Time: <2 h	N/A It might be used to detect any type of virus in body fluids, can be used for multiplexed detection, reagents can be lyophilized	[88]
Quartz-glass biochip							
CARMEN v.1	Microfluidic Cas13-based/Detect 169 human-related viruses distinguish between many influenza A subtypes of HIV and Differentiation of SARS-CoV-2 strains	NA/PCR		Fluorescence/ Y	Concentration: Reduced throughput Time: 8–10 h	Outbreak-specific Outbreak specific panels for detection of diseases should be deployed for testing of thousands of samples from a population	[89]
mCARMEN variant identification panel (VIP)	Microfluidic Cas12-based/Detect 21 viruses, including SARS-CoV-2 and influenza strains, with excellent sensitivity	NA/PCR		Fluorescence/ Y	Sensitivity: 10^2^ copies μL^−1^ Time: 8 h	N/A By integrating RVP (Respiratory Virus Panel) and VIP into a single panel need for manual work and equipment constraint can be reduced In future, the panel can be FDA approved and then commercialized	[90]
SATORI(glass)	Cas13a/SARS-CoV-2	ssRNA/Amplification free method		Fluorescence/ N	Sensitivity: ~10 fM Time: <5 min	Less sensitive than amplification-based methods (SHERLOCK, DETECTR and qPCR) In future, the SATORI-Cas12a system can be developed to performamplification-free double-stranded DNA detection	[91]
CRISPR dCas 9	dCas 9-based/SARS-CoV-2, Influenza A virus	NA/Isothermal amplification		colorimetric/ N	Sensitivity:Petamolar(pM) Time: 90 min	N/A In future, it can be used for diagnosis of drug resistant and reemerging viruses	[92]
Other detection platforms							
CAS-EXPAR	Cas 9-based/Listeria monocytogenes	NA/Isothermal amplification		Fluorescence/ N	Sensitivity: 0.82 attomolar(aM)Time: 1 h	N/A Detection of single-nucleotide mismatches and any target sequence site	[93]
CRISDA (CRISPR-Cas 9-based nicking endonuclease for initiating strand displacement amplification process)	Cas 9-based/Identification of single nucleotide polymorphisms (SNPs) and homozygous/heterozygous genotypes related to brest cancer	DNA/Isothermal amplification		Peptide nucleic acid (PNA) invasion mediated fluorescence/N	Sensitivity: aM	N/A Highly specific and sensitive detection of SNPs and targeted sequences in POCT devices	[94]
RCH (dCas9-based RCA CRISPR split HRP)	Cas 9-based/Lung cancer bycirculating let-7a biomarker and miRNA	miRNA/RCA		Chemiluminescenc/ N	Sensitivity: Femtomolar(fM) Time: <4 h	For different miRNA, a RCH probe/sgRNA isredesigned and synthesized in days Can be used as a solution or paper-based colorimetric readouts Cost can befurther reducedby industrializing the protein producing procedure. sensitivitycan be improved by implementing alternativeisothermal amplification systems or other reporting systems such as split-GFP, split-luciferase and split-β-galactosidase	[95]

### 2.7. Comparison of Various Detection Methods

A variety of methods can be used for the detection of target analytes. Commonly used optical methods for signal detection are optical cavity resonators, electrochemical impedance spectroscopy (EIS), fluorescence and surface plasmon resonance (SPR). In the SPR detection method, the refractive method is altered due to a surface based chemical reaction and causes a shift in the optical signal, whereas EIS monitors change in surface impedance due to transport or reaction on the surfaces. Apart from these, electrically based techniques utilize surface based reactions that correspond to alterations in the electrical signal such as resistance, current, capacitance or conductance of the test sample. Moreover, there are some methods that provide a signal due to the adsorption of specific molecules on cantilevers [82].

Using a microfluidic sample enrichment strategy, the PCR free LSPR (localized surface plasmon resonance) and SERS (surface enhanced Raman spectroscopy) methods have been developed for the analysis of circulating tumor nucleic acids. However, some techniques still experience false-positive results due to poor specificity of the assay, and for practical uses, it would be necessary to improve the reproducibility, specificity and analysis of the clinical samples [83]. Table 4 shows the advantages, disadvantages and future prospects of different detection methods.

### 2.8. Applications of Microfluidics

Microfluidic devices have made their contribution in innumerable applications by overcoming the difficulties of conventional assays. It has been manifested that the devices that are based on microfluidic systems contain great potential in disease diagnosis, personalized medicine, cell culture, chemical screening, cell separation, drug screening, cell treatment, drug delivery and DNA sequencing [108,109]. In disease diagnosis, microfluidic devices play a robust role in the analysis of numerous samples, which include saliva, blood and cell tissue [110]. As a microfluidic device can easily detain air borne pathogens, by simply converting the laminar flow into a twisted air flow, the probability of contact between the microfluidic channels and microbe can be increased. Moreover, hundreds of microbes are collected by the device in some microliters of the solution, which proves to be sufficient for nucleic acid or immune analyses. There are many substances that can be detected by microfluidic systems such as creatinine [111,112]. Hormones are also detected by these devices, whose most common example is the diagnosis of human chorionic gonadotropin hormone by a paper-based microfluidic device. Recent advancements have been made in this area for not only detecting but quantifying hormone levels by displaying digitally the number of weeks of pregnancy [113,114].

Nowadays, a lot of interest has been evolving in the development of organs/ tissues on chips. There are two main reasons to do this. Firstly, the experiments cannot be directly performed on humans, and secondly, human physiology cannot be imitated by animal models. Furthermore, the time required for testing and the discovery of drugs can also be reduced, along with the investment cost [110,115,116]. In drug delivery microfluidic systems, the precise dosage, controlled and sustained release of drugs, targeted delivery, likelihood of multiple dosing and emergence of very little side effects have multiple advantages over traditional drug delivery systems. Microfluidic systems have been shifted towards advanced drug delivery systems with 100% encapsulation efficiency (theoretically). Three main types of microfluidic systems for drug delivery have been used, which include drug carriers integrated with the microfluidic lab on a chip system, drug carrier-free microfluidic system and microneedle-based drug delivery systems [117,118].

**Figure 3:**
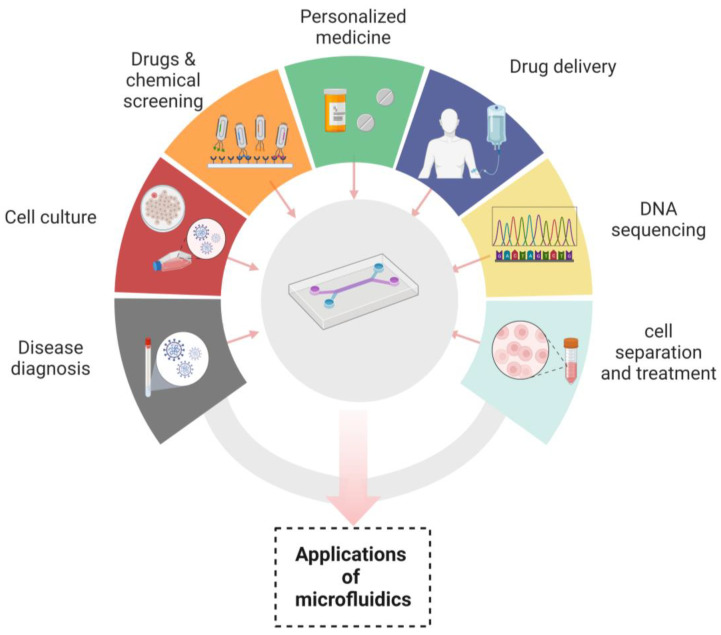
Applications of microfluidics.

The role of nanotechnology cannot be denied in revolutionizing the many aspects of medicine, drug delivery and therapeutics. Microfluidic devices have served nanotechnology in a plethora of applications, such as in the synthesis of nanoparticles. Due to their uniform shape, narrow size distribution, improved reproducibility and higher efficiency of encapsulation, microfluidic devices are excellent platforms for their synthesis. The microfluidic synthetic products that can be served as biosensors are AuNPs that differentiate the variable concentrations of *E. coli* and indicate color changes of nanoparticles through an application installed in smart phones [74]. Nanoparticles such as liposomes synthesized by microfluidic systems have been widely used in drug delivery systems because of their prolonged drug delivery and enhanced stability [119]. In addition to already available microfluidic applications, there are some distinct fields in which microfluidics have been playing their part. These are template stickers manufacturing, contact lens sensors for the assessment of the physiological parameters of astronauts, devices with low sample requirements from pediatric patients, the microfluidic machine learning based approach for processing data and making accurate predictions in results optimization. This approach has multiple advantages in next generation drug discovery, organ modeling and developmental biology [120,121]. Figure 3 Shows the applications of microfluidic systems in disease diagnosis, cell culture, chemicals and drugs screening, personalized medicines, drug delivery, DNA sequencing and cell separation and treatment. “Created with BioRender.com”.

## 3. Limits, Challenges and Policy Recommendations

### 3.1. On-Site Sample Preparation

Microfluidic POCT devices offer a rapid, easy-to-use, cost-effective approach for nucleic acid testing. At the same time, the device must be sensitive, specific and reliable in clinical settings. Microfluidic systems must be fully enclosed to avoid any contamination and evaporation of reactants. To maximize the detection efficiency, it is ideal to combine the three phases of sample preparation, nucleic acid amplification and signal detection on a single platform. However, in most cases, sample pretreatment is necessary to provide sufficient detection sensitivity. The miniaturization and standardization of the systems for on-site diagnostic purposes will be a future technological challenge. However, the more prevalent use of microfluidic devices will enable greater access to diagnostic testing for patients [122]. Therefore, there is a need for an efficient system that must be integrated on a chip for on-site sample preparation without any need for extra steps Research in the domain of microfluidic plasma separation will focus on creating such apparatuses that isolate pure and high yield plasma of greater volumes, because assays for the detection of blood biomarkers present in low quantities require large volumes of plasma.

The development of straightforward and extremely portable, user friendly devices does not require any additional hardware for these devices to be used in areas with limited resources [32,123].

### 3.2. Nonspecific Adsorption

Moreover, when there is a very low concentration of the target analyte in the sample, nonspecific adsorption of the target biomolecule to the microfluidic channel walls may result in false-negative results. Moreover, NAT assays based on the utilization of DNA probes are preferable when there is a significantly higher concentration of nucleic acid. However, nucleic acid at a low concentration is insufficient for detection by this method [13]. For that, there is a need for the modification of microfluidic channels. These modifications also play an important role in reproducibility of the results and ensure the sensitivity of the system. The capillary actions of paper-based analytical devices (µPADs) enable direct movement of the analyte from the blood or serum in the detection zone, which is accomplished without any external support. Despite the simplicity of the method and low cost, the lack of reproducibility of µPADs is a major hurdle in this regard. However, progress has been made to increase the stability of enzymes on paper by using chitosan, 3-aminopropyltriethoxysilane modified nanoparticles and multiple layers of papers or 3D-PADs.

### 3.3. ComplexityinAdopting Multiplexing Approach

For a multiplexed POCT, the optimal device should have a high sensitivity, capability of multiplexing, quick turnaround time, good sensor performance and simplicity of system. By designing different sets of primers for various targets, a multiplex DNA analysis of different targets can be performed on a single chip. Developing a single chip-based multiplexed microfluidic system has a wide range of applications, but the amplification of multiple targets in a single reactor or in multiple chambers will make the system more complex. However, there is a possibility that a better sensitivity and specificity may be obtained in this regard. Lastly, the development of a user-friendly, sensitive, specific, portable and low-cost detection method for multiplex detection will be a point of interest for research in the future.

### 3.4. Introduction of Microfluidic Devices in Wearable Systems

Although miniaturization and multiplexing have been achieved, the introduction of such techniques to wearable systems is prevented by active pumping. By skillfully combining the idea of digital microfluidics and sliding apart two plates manually, the SlipChip compartmentalizes constant fluidic paths into discrete volumes without the use of external pumps. The same group reported a thirty minute digital antibiotic sensitivity test on a urine sample based on SlipChip [124,125]. Utilizing vacuum chambers is a smart replacement for the use of external pumps. Two significant challenges are faced during the designing of wearable sensors for very low level biomarker detection: wash free and label-free assays optimized for the integration of systems and the rate of recognition site regeneration for constant monitoring [126].

### 3.5. Signal Readout

A semi quantitative readout signal is another drawback in the paper-based microfluidic approach, which can be improved by using alternative detection methods such as nanodots or electrochemistry-based fluorescence detection that can improve the quantification of PAD. This underlines the conflict between the usefulness and simplicity of PADs, because this strategy necessitates external support, which would undermine their portability and usability.

In recent years, there have been numerous reports on monolithic microfluidic devices that perform sample preparation, isolation, amplification and detection on a single platform. Therefore, we anticipate additional development in integrated systems, such as multiplexed testing for the simultaneous detection of multiple analytes. In the future, the commercialization of such devices will necessitate the mass production of disposable chips at reasonable rates. Two requirements should be considered to achieve this goal: inexpensive and disposable polymers that will be preferable for mass production and will have sufficient detection characteristics. However, the materials must possess enough thermal and mechanical qualities to withstand the temperatures needed for the amplification of nucleic acids [127,128,129,130,131,132].

## 4. Conclusions

The isothermal amplification of nucleic acids using micro-structured microfluidic devices demonstrates a significant ability for the greater speed and cost-effective automation of procedures ranging from sample preparation to signal detection. A lower sample volume requirement is quite helpful, especially when less amounts of samples are available. Nucleic acids can be extracted and amplified within minutes to hours on a microfluidic device without the use of conventional burdensome sample preparation and amplification processes by merging sample preparation and one of the methods for isothermal amplification. The utilization of modular microfluidic technology has made it possible to design a microfluidic device according to one’s requirements by selecting a module and connection method of your own choice. Signal detection is also performed on-chip, which makes it a convenient and an ideal POCT device. Due to better sensitivity and specificity, NAT assays have prevailed over the use of conventional immunoassays. The selection of the amplification type for microfluidic NAT entirely depends upon the nature of the target. Despite its inception, the field of microfluidics has attained attention from researchers present all over the world.

Recent advancements in CRISPR-based detection systems have drawn researchers’ interest in the detection of individual and multiple diseases on a single platform. By using CRISPR/Cas technology in microfluidic systems, next-generation diagnostic methods can be improved to a certain extent. The contribution of the microfluidics integrated fluid mechanics approach for the synthesis of nanoparticles with homogenous sizes and shapes has been utilized in various applications of the biosciences and health care sectors. Conventional procedures need huge instrumentation, extra power, heat loss and plenty of time for the synthesis of nanoparticles, whereas traditional tools have been reduced to one platform for micro and nanoparticle syntheses. The nanoparticles synthesized by microfluidics allow fast handling and better efficacy of the procedure using the smallest components to run the process. Moreover, microfluidic technology has benefited the biomedicine field by proving itself a suitable candidate for the diagnosis of diseases, observing pandemics and glucose monitoring. However, it is anticipated that the discipline will increase the understanding of biomedicine, nanoparticle synthesis and point towards interruptions by addressing numerous healthcare related issues.

## Figures and Tables

**Table 1:** Comparison of microfluidic approaches for serum/ plasma separation.

MicrofluidicApproach	Mechanism	Sample	Target	Material	Ref.
Sedimentation	Sedimentation	Blood	Plasma	Glass	[28]
Microfiltration	Sedimentation	Blood	Plasma	/PDMS/PTFE/Glass	[29]
Lateral displacement	Filtration	Blood	Plasma	PMMA	[30]
Hydrodynamic	Hydrodynamic	Blood	Plasma	PDMS	[31]
Acoustic separation	Dielectrophoretic Hydrodynamic Acoustic	Blood	Plasma	PDMS	[32]
Dielectrophoresis	Dielectrophoretic Hydrodynamic Acoustic	Blood	Plasma	PDMS	[33]
Compact Disc (CD) format	Sedimentation	Blood	Plasma	COC/PDMS/pMMA	[34]

**Table 2:** Comparison of isothermal methods for nucleic acid amplification.

Isothermal Method	Template	Time	Primers	Tm (°C)	Ref.
LAMP	DNA/RNA	15–60 min	3 pairs	60–65	[59,60]
HDA	DNA, rRNA	1–1.5 h	1 pair	60–65	[61,62]
RCA	cssDNA, RNA, miRNA	1 h	1 single primer, 1 padlock probe	25–37	[63,64]
MDA	dsDNA	2 h	Random hexamer	30	[65,66]
RPA	DNA/RNA	5–7 min	1 pair	37–42	[60,61,62]
NASBA	SsRNA, tmRNA, rRNA ^1^	1.5 h	1 pair	41	[63,64]

^1^ SsRNA: Single-stranded RNA, tmRNA: transfer–messenger RNA, rRNA: ribosomal RNA, dsDNA: double-stranded DNA, miRNA: microRNA, cssDNA: circular single-stranded DNA, Tm: melting temperature.

**Table 4:** Prospects of the detection methods.

Detection Method	Advantages	Disadvantages	Future Prospects	Refs.
Colorimetric	Visualized detection, simplicity	Limited sensitivity and no quantitative detection	In nanoparticle-based colorimetric assays, sensitivity can be increased by increasing size of nanoparticle and by decreasing density of receptor group present on surface of nanoparticle	[84,85,86]
Electrochemical	Low cost, robust response, high sensitivity	Interference susceptibility and weak stability	Biocompatibility of nanoparticles can lead to reduction in toxicity detection as the nanoparticles are less reactive against proteins	[87,88,89,90,91,92,93]
Chemiluminescence	Simplicity, high sensitivity	Enzyme dependent and time consuming	Designing a multiplex system with increased sample throughput. Quantitative approach should bead opted in future	[89]
Fluorescence	Experimental simplicity, flexibility and robust response	Background with a high fluorescence	Development of biosensors based on macro and micromolecule imprinting technology can be considered. Reusable devices based on the green chemistry approach can be designed	[94,95,96,97]
Magnetic	Detection of high signal to noise ratio, low cost	Limited availability of miniaturized magnetic readout systems	Integration in point of care testing devices	[98,99,100]
SPR-based	Label-free, real-time detection	Expensive, bulky equipment Low limit of detection	Improvement in sensitivity and detection capability Different shapes of nanoparticles can be used to improve sensitivity	[101,102,103]
SERS-based	Low background, no photobleaching, good multiplexing capabilities and high sensitivity	Limited view field, Difficulty in fabrication of SERS active substrate in microfluidic chip	Improvement in reproducibility, selectivity, integration of chip and multi-functionality	[104,105,106,107]

## Data Availability

Not applicable.

## References

[1] Stockmaier S., Stroeymeyt N., Shattuck E.C., Hawley D.M., Meyers L.A., Bolnick D.I. (2021). Infectious Diseases and Social Distancing in Nature. Science.

[2] Funk S., Salathé M., Jansen V.A.A. (2010). Modelling the Influence of Human Behaviour on the Spread of Infectious Diseases: A Review. J. R. Soc. Interface.

[3] Khan S.A., Ahmad I., Khan H., Abdullah, Akbar S. (2022). The Contagious Nature of SARS-CoV-2 Omicron Variant and Vaccine Efficacy. Adv. Life Sci..

[4] Lazcka O., Campo F.J.D., Muñoz F.X. (2007). Pathogen Detection: A Perspective of Traditional Methods and Biosensors. Biosens. Bioelectron..

[5] Rajakaruna S.J., Liu W.-B., Ding Y.-B., Cao G.-W. (2017). Strategy and Technology to Prevent Hospital-Acquired Infections: Lessons from SARS, Ebola, and MERS in Asia and West Africa. Mil. Med. Res..

[6] Stone H.A., Stroock A.D., Ajdari A. (2004). Engineering Flows in Small Devices: Microfluidics Toward a Lab-on-a-Chip. Annu. Rev. Fluid Mech..

[7] Cavaniol C., Cesar W., Descroix S., Viovy J.-L. (2022). Flowmetering for Microfluidics. Lab Chip.

[8] Xu H., Xia A., Wang D., Zhang Y., Deng S., Lu W., Luo J., Zhong Q., Zhang F., Zhou L. (2020). An Ultraportable and Versatile Point-of-Care DNA Testing Platform. Sci. Adv..

[9] Ortseifen V., Viefhues M., Wobbe L., Grünberger A. (2020). Microfluidics for Biotechnology: Bridging Gaps to Foster Microfluidic Applications. Front. Bioeng. Biotechnol..

[10] Wang X., Hong X.-Z., Li Y.-W., Li Y., Wang J., Chen P., Liu B.-F. (2022). Microfluidics-Based Strategies for Molecular Diagnostics of Infectious Diseases. Mil. Med. Res..

[11] Tarim E.A., Karakuzu B., Oksuz C., Sarigil O., Kizilkaya M., Al-Ruweidi M.K.A.A., Yalcin H.C., Ozcivici E., Tekin H.C. (2021). Microfluidic-Based Virus Detection Methods for Respiratory Diseases. Emergent Mater..

[12] Lui C., Cady N., Batt C. (2009). Nucleic Acid-Based Detection of Bacterial Pathogens Using Integrated Microfluidic Platform Systems. Sensors.

[13] Silbeglitt R., Wong A. (2009). The Global Technology Revolution China, In-Depth Analyses: Emerging Technology Opportunities for the Tianjin Binhai New Area (TBNA) and the Tianjin Technological Development Area (TEDA).

[14] Yeh E.-C., Fu C.-C., Hu L., Thakur R., Feng J., Lee L.P. (2017). Self-Powered Integrated Microfluidic Point-of-Care Low-Cost Enabling (SIMPLE) Chip. Sci. Adv..

[15] Carrell C., Kava A., Nguyen M., Menger R., Munshi Z., Call Z., Nussbaum M., Henry C. (2019). Beyond the Lateral Flow Assay: A Review of Paper-Based Microfluidics. Microelectron. Eng..

[16] Fraser L.A., Cheung Y., Kinghorn A.B., Guo W., Shiu S.C., Jinata C., Liu M., Bhuyan S., Nan L., Shum H.C. (2019). Microfluidic Technology for Nucleic Acid Aptamer Evolution and Application. Adv. Biosys..

[17] Ye X., Xu J., Lu L., Li X., Fang X., Kong J. (2018). Equipment-Free Nucleic Acid Extraction and Amplification on a Simple Paper Disc for Point-of-Care Diagnosis of Rotavirus A. Anal. Chim. Acta.

[18] Wu J., Fang H., Zhang J., Yan S. (2023). Modular Microfluidics for Life Sciences. J. Nanobiotechnol..

[19] Urbaniak J., Janowski D., Jacewski B. (2019). Isolation of Nucleic Acids Using Silicon Dioxide Powder as a Tool for Environmental Monitoring. Environ. Monit. Assess..

[20] Watanabe R., Asai S., Kakizoe H., Saeki H., Masukawa A., Miyazawa M., Ohtagawa K., Ravzanaaadii M.-A., Doi M., Atsumi H. (2021). Evaluation of the Basic Assay Performance of the GeneSoc^®^ Rapid PCR Testing System for Detection of Severe Acute Respiratory Syndrome Coronavirus 2. PLoS ONE.

[21] Dong X., Liu L., Tu Y., Zhang J., Miao G., Zhang L., Ge S., Xia N., Yu D., Qiu X. (2021). Rapid PCR Powered by Microfluidics: A Quick Review under the Background of COVID-19 Pandemic. TrAC Trends Anal. Chem..

[22] Mendoza-Gallegos R.A., Rios A., Garcia-Cordero J.L. (2018). An Affordable and Portable Thermocycler for Real-Time PCR Made of 3D-Printed Parts and Off-the-Shelf Electronics. Anal. Chem..

[23] Jafek A.R., Harbertson S., Brady H., Samuel R., Gale B.K. (2018). Instrumentation for XPCR Incorporating QPCR and HRMA. Anal. Chem..

[24] Espulgar W.V., Saito M., Takahashi K., Ushiro S., Yamamoto N., Akeda Y., Hamaguchi S., Tomono K., Tamiya E. (2021). Deskilled and Rapid Drug-Resistant Gene Detection by Centrifugal Force-Assisted Thermal Convection PCR Device. Sensors.

[25] Nge P.N., Rogers C.I., Woolley A.T. (2013). Advances in Microfluidic Materials, Functions, Integration, and Applications. Chem. Rev..

[26] Wei X., Tian T., Jia S., Zhu Z., Ma Y., Sun J., Lin Z., Yang C.J. (2015). Target-Responsive DNA Hydrogel Mediated “Stop-Flow” Microfluidic Paper-Based Analytic Device for Rapid, Portable and Visual Detection of Multiple Targets. Anal. Chem..

[27] Hu J., Xiao K., Jin B., Zheng X., Ji F., Bai D. (2019). Paper-based Point-of-care Test with Xeno Nucleic Acid Probes. Biotechnol. Bioeng..

[28] Chavez-Pineda O.G., Rodriguez-Moncayo R., Cedillo-Alcantar D.F., Guevara-Pantoja P.E., Amador-Hernandez J.U., Garcia-Cordero J.L. (2022). Microfluidic Systems for the Analysis of Blood-derived Molecular Biomarkers. Electrophoresis.

[29] Sun M., Khan Z.S., Vanapalli S.A. (2012). Blood Plasma Separation in a Long Two-Phase Plug Flowing through Disposable Tubing. Lab Chip.

[30] Zhang X.-B., Wu Z.-Q., Wang K., Zhu J., Xu J.-J., Xia X.-H., Chen H.-Y. (2012). Gravitational Sedimentation Induced Blood Delamination for Continuous Plasma Separation on a Microfluidics Chip. Anal. Chem..

[31] Gao Q., Chang Y., Deng Q., You H. (2020). A Simple and Rapid Method for Blood Plasma Separation Driven by Capillary Force with an Application in Protein Detection. Anal. Methods.

[32] Jiang F., Xiang N., Ni Z. (2020). Ultrahigh Throughput Beehive-like Device for Blood Plasma Separation. Electrophoresis.

[33] Szydzik C., Khoshmanesh K., Mitchell A., Karnutsch C. (2015). Microfluidic Platform for Separation and Extraction of Plasma from Whole Blood Using Dielectrophoresis. Biomicrofluidics.

[34] Lenshof A., Ahmad-Tajudin A., Järås K., Swärd-Nilsson A.-M., Åberg L., Marko-Varga G., Malm J., Lilja H., Laurell T. (2009). Acoustic Whole Blood Plasmapheresis Chip for Prostate Specific Antigen Microarray Diagnostics. Anal. Chem..

[35] Wilson C.J., Clegg R.E., Leavesley D.I., Pearcy M.J. (2005). Mediation of Biomaterial–Cell Interactions by Adsorbed Proteins: A Review. Tissue Eng..

[36] Sposito A., Hoang V., DeVoe D.L. (2016). Rapid Real-Time PCR and High Resolution Melt Analysis in a Self-Filling Thermoplastic Chip. Lab Chip.

[37] Kim T.-H., Sunkara V., Park J., Kim C.-J., Woo H.-K., Cho Y.-K. (2016). A Lab-on-a-Disc with Reversible and Thermally Stable Diaphragm Valves. Lab Chip.

[38] Houssin T., Cramer J., Grojsman R., Bellahsene L., Colas G., Moulet H., Minnella W., Pannetier C., Leberre M., Plecis A. (2016). Ultrafast, Sensitive and Large-Volume on-Chip Real-Time PCR for the Molecular Diagnosis of Bacterial and Viral Infections. Lab Chip.

[39] Roy E., Stewart G., Mounier M., Malic L., Peytavi R., Clime L., Madou M., Bossinot M., Bergeron M.G., Veres T. (2015). From Cellular Lysis to Microarray Detection, an Integrated Thermoplastic Elastomer (TPE) Point of Care Lab on a Disc. Lab Chip.

[40] Shin D.J., Trick A.Y., Hsieh Y.-H., Thomas D.L., Wang T.-H. (2018). Sample-to-Answer Droplet Magnetofluidic Platform for Point-of-Care Hepatitis C Viral Load Quantitation. Sci. Rep..

[41] Zanoli L., Spoto G. (2012). Isothermal Amplification Methods for the Detection of Nucleic Acids in Microfluidic Devices. Biosensors.

[42] Lee S.-Y., Huang J.-G., Chuang T.-L., Sheu J.-C., Chuang Y.-K., Holl M., Meldrum D.R., Lee C.-N., Lin C.-W. (2008). Compact Optical Diagnostic Device for Isothermal Nucleic Acids Amplification. Sens. Actuators B Chem..

[43] Lee S.-Y., Lee C.-N., Mark H., Meldrum D.R., Lin C.-W. (2007). Efficient, Specific, Compact Hepatitis B Diagnostic Device: Optical Detection of the Hepatitis B Virus by Isothermal Amplification. Sens. Actuators B Chem..

[44] Ding X., Wang G., Mu Y. (2019). Single Enzyme-Based Stem-Loop and Linear Primers Co-Mediated Exponential Amplification of Short Gene Sequences. Anal. Chim. Acta.

[45] Jeong Y.-J., Park K., Kim D.-E. (2009). Isothermal DNA Amplification in Vitro: The Helicase-Dependent Amplification System. Cell. Mol. Life Sci..

[46] Mahalanabis M., Do J., ALMuayad H., Zhang J.Y., Klapperich C.M. (2010). An Integrated Disposable Device for DNA Extraction and Helicase Dependent Amplification. Biomed. Microdevices.

[47] Kolm C., Martzy R., Führer M., Mach R.L., Krska R., Baumgartner S., Farnleitner A.H., Reischer G.H. (2019). Detection of a Microbial Source Tracking Marker by Isothermal Helicase-Dependent Amplification and a Nucleic Acid Lateral-Flow Strip Test. Sci. Rep..

[48] Mothershed E.A., Whitney A.M. (2006). Nucleic Acid-Based Methods for the Detection of Bacterial Pathogens: Present and Future Considerations for the Clinical Laboratory. Clin. Chim. Acta.

[49] Huang R., Di K., Adeel K., Fan B., Gu X., Xu H., Shen H., He N., Li Z. (2022). The Exploration of Droplet Digital Branched Rolling Circle Amplification Based Ultrasensitive Biosensor for Gastric Cancer Cell-Derived Extracellular Vesicles Detection. Mater. Today Adv..

[50] Hashkavayi A.B., Cha B.S., Lee E.S., Park K.S. (2022). Dual Rolling Circle Amplification-Enabled Ultrasensitive Multiplex Detection of Exosome Biomarkers Using Electrochemical Aptasensors. Anal. Chim. Acta.

[51] Marcy Y., Ishoey T., Lasken R.S., Stockwell T.B., Walenz B.P., Halpern A.L., Beeson K.Y., Goldberg S.M.D., Quake S.R. (2007). Nanoliter Reactors Improve Multiple Displacement Amplification of Genomes from Single Cells. PLoS Genet..

[52] Dean F.B., Hosono S., Fang L., Wu X., Faruqi A.F., Bray-Ward P., Sun Z., Zong Q., Du Y., Du J. (2002). Comprehensive Human Genome Amplification Using Multiple Displacement Amplification. Proc. Natl. Acad. Sci. USA.

[53] Zhang K., Martiny A.C., Reppas N.B., Barry K.W., Malek J., Chisholm S.W., Church G.M. (2006). Sequencing Genomes from Single Cells by Polymerase Cloning. Nat. Biotechnol..

[54] Wu N., Zhang Y., Fu J., Zhang R., Feng L., Hu Y., Li X., Lu N., Zhao X., Pan Y. (2012). Performance Assessment of a Novel Two-Step Multiple Displacement Amplification-PCR Assay for Detection of Mycobacterium Tuberculosis Complex in Sputum Specimens. J. Clin. Microbiol..

[55] Hakenberg S., Hügle M., Weidmann M., Hufert F., Dame G., Urban G.A. (2012). A Phaseguided Passive Batch Microfluidic Mixing Chamber for Isothermal Amplification. Lab Chip.

[56] Kersting S., Rausch V., Bier F.F., von Nickisch-Rosenegk M. (2014). Multiplex Isothermal Solid-Phase Recombinase Polymerase Amplification for the Specific and Fast DNA-Based Detection of Three Bacterial Pathogens. Microchim. Acta.

[57] Alamolhoda S.Z., Zarghami N., Kahroba H., Mehdipour A., Pourhassan-Moghaddam M., Jahanban-Esfahlan R., Milani M. (2021). Isothermal Amplification of Nucleic Acids Coupled with Nanotechnology and Microfluidic Platforms for Detecting Antimicrobial Drug Resistance and Beyond. Adv. Pharm. Bull..

[58] Zhao X., Dong T., Yang Z., Pires N., Høivik N. (2012). Compatible Immuno-NASBA LOC Device for Quantitative Detection of Waterborne Pathogens: Design and Validation. Lab Chip.

[59] Hardinge P., Murray J.A.H. (2019). Reduced False Positives and Improved Reporting of Loop-Mediated Isothermal Amplification Using Quenched Fluorescent Primers. Sci. Rep..

[60] Dolka B., Cisek A.A., Szeleszczuk P. (2019). The Application of the Loop-Mediated Isothermal Amplification (LAMP) Method for Diagnosing Enterococcus Hirae-Associated Endocarditis Outbreaks in Chickens. BMC Microbiol..

[61] Barreda-García S., Miranda-Castro R., de-los-Santos-Álvarez N., Miranda-Ordieres A.J., Lobo-Castañón M.J. (2018). Helicase-Dependent Isothermal Amplification: A Novel Tool in the Development of Molecular-Based Analytical Systems for Rapid Pathogen Detection. Anal. Bioanal. Chem..

[62] Cao Y., Kim H., Li Y., Kong H., Lemieux B. (2013). Helicase-Dependent Amplification of Nucleic Acids. Curr. Protoc. Mol. Biol..

[63] Murakami T., Sumaoka J., Komiyama M. (2009). Sensitive Isothermal Detection of Nucleic-Acid Sequence by Primer Generation–Rolling Circle Amplification. Nucleic Acids Res..

[64] Pumford E.A., Lu J., Spaczai I., Prasetyo M.E., Zheng E.M., Zhang H., Kamei D.T. (2020). Developments in Integrating Nucleic Acid Isothermal Amplification and Detection Systems for Point-of-Care Diagnostics. Biosens. Bioelectron..

[65] Spits C., Le Caignec C., De Rycke M., Van Haute L., Van Steirteghem A., Liebaers I., Sermon K. (2006). Whole-Genome Multiple Displacement Amplification from Single Cells. Nat. Protoc..

[66] Bleier S., Maier P., Allgayer H., Wenz F., Zeller W.J., Fruehauf S., Laufs S. (2008). Multiple Displacement Amplification Enables Large-Scale Clonal Analysis Following Retroviral Gene Therapy. J. Virol..

[67] Draz U., Ali S., Firyal S., Ali A., Saleem A.H., Tahir M.A. (2019). Detection of Human Salivary Amylase Level Deposited on Fruits with First Bite Mark. Adv. Life Sci..

[68] Mauk M.G., Liu C., Sadik M., Bau H.H., Rasooly A., Herold K.E. (2015). Microfluidic Devices for Nucleic Acid (NA) Isolation, Isothermal NA Amplification, and Real-Time Detection. Mobile Health Technologies.

[69] Berkenbrock J.A., Grecco-Machado R., Achenbach S. (2020). Microfluidic Devices for the Detection of Viruses: Aspects of Emergency Fabrication during the COVID-19 Pandemic and Other Outbreaks. Proc. R. Soc. A.

[70] Fang X., Liu Y., Kong J., Jiang X. (2010). Loop-Mediated Isothermal Amplification Integrated on Microfluidic Chips for Point-of-Care Quantitative Detection of Pathogens. Anal. Chem..

[71] Klausner J.D. (2004). Editorial Commentary: The NAAT Is Out of the Bag. Clin. Infect. Dis..

[72] Hadgu A., Dendukuri N., Hilden J. (2005). Evaluation of Nucleic Acid Amplification Tests in the Absence of a Perfect Gold-Standard Test: A Review of the Statistical and Epidemiologic Issues. Epidemiology.

[73] Xie Y., Li H., Chen F., Udayakumar S., Arora K., Chen H., Lan Y., Hu Q., Zhou X., Guo X. (2022). Clustered Regularly Interspaced Short Palindromic Repeats-Based Microfluidic System in Infectious Diseases Diagnosis: Current Status, Challenges, and Perspectives. Adv. Sci..

[74] Zheng C., Wang K., Zheng W., Cheng Y., Li T., Cao B., Jin Q., Cui D. (2021). Rapid Developments in Lateral Flow Immunoassay for Nucleic Acid Detection. Analyst.

[75] Hu J., Wang L., Li F., Han Y.L., Lin M., Lu T.J., Xu F. (2013). Oligonucleotide-Linked Gold Nanoparticle Aggregates for Enhanced Sensitivity in Lateral Flow Assays. Lab Chip.

[76] Hill H.D., Mirkin C.A. (2006). The Bio-Barcode Assay for the Detection of Protein and Nucleic Acid Targets Using DTT-Induced Ligand Exchange. Nat. Protoc..

[77] Yamada K., Henares T.G., Suzuki K., Citterio D. (2015). Paper-Based Inkjet-Printed Microfluidic Analytical Devices. Angew. Chem. Int. Ed..

[78] Yabuta T., Bescher E.P., Mackenzie J.D., Tsuru K., Hayakawa S., Osaka A. (2003). Synthesis of PDMS-Based Porous Materials for Biomedical Applications. J. Sol-Gel Sci. Technol..

[79] Musgrave C.S.A., Fang F. (2019). Contact Lens Materials: A Materials Science Perspective. Materials.

[80] Liong M., Hoang A.N., Chung J., Gural N., Ford C.B., Min C., Shah R.R., Ahmad R., Fernandez-Suarez M., Fortune S.M. (2013). Magnetic Barcode Assay for Genetic Detection of Pathogens. Nat. Commun..

[81] Ng A.H.C., Fobel R., Fobel C., Lamanna J., Rackus D.G., Summers A., Dixon C., Dryden M.D.M., Lam C., Ho M. (2018). A Digital Microfluidic System for Serological Immunoassays in Remote Settings. Sci. Transl. Med..

[82] Qavi A.J., Washburn A.L., Byeon J.-Y., Bailey R.C. (2009). Label-Free Technologies for Quantitative Multiparameter Biological Analysis. Anal. Bioanal. Chem..

[83] Das J., Kelley S.O. (2020). High-Performance Nucleic Acid Sensors for Liquid Biopsy Applications. Angew. Chem. Int. Ed..

[84] Wu K., Liu J., Saha R., Su D., Krishna V.D., Cheeran M.C.-J., Wang J.-P. (2020). Magnetic Particle Spectroscopy for Detection of Influenza A Virus Subtype H1N1. ACS Appl. Mater. Interfaces.

[85] Cesewski E., Johnson B.N. (2020). Electrochemical Biosensors for Pathogen Detection. Biosens. Bioelectron..

[86] Zhang Y., McKelvie I.D., Cattrall R.W., Kolev S.D. (2016). Colorimetric Detection Based on Localised Surface Plasmon Resonance of Gold Nanoparticles: Merits, Inherent Shortcomings and Future Prospects. Talanta.

[87] Wongkaew N., Simsek M., Griesche C., Baeumner A.J. (2019). Functional Nanomaterials and Nanostructures Enhancing Electrochemical Biosensors and Lab-on-a-Chip Performances: Recent Progress, Applications, and Future Perspective. Chem. Rev..

[88] Reta N., Saint C.P., Michelmore A., Prieto-Simon B., Voelcker N.H. (2018). Nanostructured Electrochemical Biosensors for Label-Free Detection of Water- and Food-Borne Pathogens. ACS Appl. Mater. Interfaces.

[89] Sun Y., Lu J. (2018). Chemiluminescence-Based Aptasensors for Various Target Analytes. Luminescence.

[90] Xiao Q., Xu C. (2020). Research Progress on Chemiluminescence Immunoassay Combined with Novel Technologies. TrAC Trends Anal. Chem..

[91] Al Mughairy B., Al-Lawati H.A.J. (2020). Recent Analytical Advancements in Microfluidics Using Chemiluminescence Detection Systems for Food Analysis. TrAC Trends Anal. Chem..

[92] Liu M., Khan A., Wang Z., Liu Y., Yang G., Deng Y., He N. (2019). Aptasensors for Pesticide Detection. Biosens. Bioelectron..

[93] Singh P., Pandey S.K., Singh J., Srivastava S., Sachan S., Singh S.K. (2016). Biomedical Perspective of Electrochemical Nanobiosensor. Nano-Micro Lett..

[94] Ansari S., Masoum S. (2021). Recent Advances and Future Trends on Molecularly Imprinted Polymer-Based Fluorescence Sensors with Luminescent Carbon Dots. Talanta.

[95] Jiang P., Guo Z. (2004). Fluorescent Detection of Zinc in Biological Systems: Recent Development on the Design of Chemosensors and Biosensors. Coord. Chem. Rev..

[96] VanEngelenburg S.B., Palmer A.E. (2008). Fluorescent Biosensors of Protein Function. Curr. Opin. Chem. Biol..

[97] Pazos E., Vázquez O., Mascareñas J.L., Eugenio Vázquez M. (2009). Peptide-Based Fluorescent Biosensors. Chem. Soc. Rev..

[98] Denmark D.J., Bustos-Perez X., Swain A., Phan M.-H., Mohapatra S., Mohapatra S.S. (2019). Readiness of Magnetic Nanobiosensors for Point-of-Care Commercialization. J. Elec. Mater..

[99] Xianyu Y., Wang Q., Chen Y. (2018). Magnetic Particles-Enabled Biosensors for Point-of-Care Testing. TrAC Trends Anal. Chem..

[100] Pashchenko O., Shelby T., Banerjee T., Santra S. (2018). A Comparison of Optical, Electrochemical, Magnetic, and Colorimetric Point-of-Care Biosensors for Infectious Disease Diagnosis. ACS Infect. Dis..

[101] Zhou J., Qi Q., Wang C., Qian Y., Liu G., Wang Y., Fu L. (2019). Surface Plasmon Resonance (SPR) Biosensors for Food Allergen Detection in Food Matrices. Biosens. Bioelectron..

[102] Mahmoudpour M., Ezzati Nazhad Dolatabadi J., Torbati M., Pirpour Tazehkand A., Homayouni-Rad A., de la Guardia M. (2019). Nanomaterials and New Biorecognition Molecules Based Surface Plasmon Resonance Biosensors for Mycotoxin Detection. Biosens. Bioelectron..

[103] Sepúlveda B., Angelomé P.C., Lechuga L.M., Liz-Marzán L.M. (2009). LSPR-Based Nanobiosensors. Nano Today.

[104] Guo J., Zeng F., Guo J., Ma X. (2020). Preparation and Application of Microfluidic SERS Substrate: Challenges and Future Perspectives. J. Mater. Sci. Technol..

[105] Vendrell M., Maiti K.K., Dhaliwal K., Chang Y.-T. (2013). Surface-Enhanced Raman Scattering in Cancer Detection and Imaging. Trends Biotechnol..

[106] Langer J., Jimenez de Aberasturi D., Aizpurua J., Alvarez-Puebla R.A., Auguié B., Baumberg J.J., Bazan G.C., Bell S.E.J., Boisen A., Brolo A.G. (2020). Present and Future of Surface-Enhanced Raman Scattering. ACS Nano.

[107] Tu Q., Chang C. (2012). Diagnostic Applications of Raman Spectroscopy. Nanomed. Nanotechnol. Biol. Med..

[108] Sengupta P., Khanra K., Chowdhury A.R., Datta P. (2019). Lab-on-a-Chip Sensing Devices for Biomedical Applications. Bioelectronics and Medical Devices.

[109] Nielsen J.B., Hanson R.L., Almughamsi H.M., Pang C., Fish T.R., Woolley A.T. (2020). Microfluidics: Innovations in Materials and Their Fabrication and Functionalization. Anal. Chem..

[110] Merrin J. (2019). Frontiers in Microfluidics, a Teaching Resource Review. Bioengineering.

[111] Jing W., Sui G., Wang C., Leblanc R.M. (2015). Bioanalysis within Microfluidics: A Review. ACS Symposium Series.

[112] Narimani R., Azizi M., Esmaeili M., Rasta S.H., Khosroshahi H.T. (2020). An Optimal Method for Measuring Biomarkers: Colorimetric Optical Image Processing for Determination of Creatinine Concentration Using Silver Nanoparticles. 3 Biotech..

[113] Walgama C., Nguyen M.P., Boatner L.M., Richards I., Crooks R.M. (2020). Hybrid Paper and 3D-Printed Microfluidic Device for Electrochemical Detection of Ag Nanoparticle Labels. Lab Chip.

[114] Gnoth C., Johnson S. (2014). Strips of Hope: Accuracy of Home Pregnancy Tests and New Developments. Geburtshilfe Frauenheilkd.

[115] Oh K.W. (2020). Microfluidic Devices for Biomedical Applications: Biomedical Microfluidic Devices 2019. Micromachines.

[116] Williams M.J., Lee N.K., Mylott J.A., Mazzola N., Ahmed A., Abhyankar V.V. (2019). A Low-Cost, Rapidly Integrated Debubbler (RID) Module for Microfluidic Cell Culture Applications. Micromachines.

[117] Sanjay S.T., Zhou W., Dou M., Tavakoli H., Ma L., Xu F., Li X. (2018). Recent Advances of Controlled Drug Delivery Using Microfluidic Platforms. Adv. Drug Deliv. Rev..

[118] Martins J.P., Torrieri G., Santos H.A. (2018). The Importance of Microfluidics for the Preparation of Nanoparticles as Advanced Drug Delivery Systems. Expert Opin. Drug Deliv..

[119] Arduino I., Liu Z., Rahikkala A., Figueiredo P., Correia A., Cutrignelli A., Denora N., Santos H.A. (2021). Preparation of Cetyl Palmitate-Based PEGylated Solid Lipid Nanoparticles by Microfluidic Technique. Acta Biomater..

[120] Galan E.A., Zhao H., Wang X., Dai Q., Huck W.T.S., Ma S. (2020). Intelligent Microfluidics: The Convergence of Machine Learning and Microfluidics in Materials Science and Biomedicine. Matter.

[121] Niculescu A.-G., Chircov C., Bîrcă A.C., Grumezescu A.M. (2021). Fabrication and Applications of Microfluidic Devices: A Review. Int. J. Mol. Sci..

[122] Wang D., Chan H.N., Liu Z., Micheal S., Li L., Baniani D.B., Tan M.J.A., Huang L., Wang J., Wu H., Jiang X., Bai C., Liu M. (2020). Recent Developments in Microfluidic-Based Point-of-care Testing (POCT) Diagnoses. Nanotechnology and Microfluidics.

[123] Liu Y.-Y., Wang Y., Walsh T.R., Yi L.-X., Zhang R., Spencer J., Doi Y., Tian G., Dong B., Huang X. (2016). Emergence of Plasmid-Mediated Colistin Resistance Mechanism MCR-1 in Animals and Human Beings in China: A Microbiological and Molecular Biological Study. Lancet Infect. Dis..

[124] Shen F., Du W., Kreutz J.E., Fok A., Ismagilov R.F. (2010). Digital PCR on a SlipChip. Lab Chip.

[125] Schoepp N.G., Schlappi T.S., Curtis M.S., Butkovich S.S., Miller S., Humphries R.M., Ismagilov R.F. (2017). Rapid Pathogen-Specific Phenotypic Antibiotic Susceptibility Testing Using Digital LAMP Quantification in Clinical Samples. Sci. Transl. Med..

[126] Tu J., Torrente-Rodríguez R.M., Wang M., Gao W. (2020). The Era of Digital Health: A Review of Portable and Wearable Affinity Biosensors. Adv. Funct. Mater..

[127] Lee S., Oncescu V., Mancuso M., Mehta S., Erickson D. (2014). A Smartphone Platform for the Quantification of Vitamin D Levels. Lab Chip.

[128] Zhu W.-J., Feng D.-Q., Chen M., Chen Z.-D., Zhu R., Fang H.-L., Wang W. (2014). Bienzyme Colorimetric Detection of Glucose with Self-Calibration Based on Tree-Shaped Paper Strip. Sens. Actuators B Chem..

[129] Park C., Han Y.D., Kim H.V., Lee J., Yoon H.C., Park S. (2018). Double-Sided 3D Printing on Paper towards Mass Production of Three-Dimensional Paper-Based Microfluidic Analytical Devices (3D-ΜPADs). Lab Chip.

[130] Parween S., Subudhi P.D., Asthana A. (2019). An Affordable, Rapid Determination of Total Lipid Profile Using Paper-Based Microfluidic Device. Sens. Actuators B Chem..

[131] Li F., Wang X., Liu J., Hu Y., He J. (2019). Double-Layered Microfluidic Paper-Based Device with Multiple Colorimetric Indicators for Multiplexed Detection of Biomolecules. Sens. Actuators B Chem..

[132] Chen X., Chen J., Wang F., Xiang X., Luo M., Ji X., He Z. (2012). Determination of Glucose and Uric Acid with Bienzyme Colorimetry on Microfluidic Paper-Based Analysis Devices. Biosens. Bioelectron..

